# Mapping ethical issues in the use of smart home health technologies to care for older persons: a systematic review

**DOI:** 10.1186/s12910-023-00898-w

**Published:** 2023-03-29

**Authors:** Nadine Andrea Felber, Yi Jiao (Angelina) Tian, Félix Pageau, Bernice Simone Elger, Tenzin Wangmo

**Affiliations:** 1 Institute of Biomedical Ethics, University of Basel, Bernoullistrasse 28, 4056 Basel, Switzerland; 2 Faculty of Medicine, Université Laval, 1050 Av. de la Médecine, G1V0A6, Québec, QC Canada

**Keywords:** Biomedical ethics, Caregiving, Older persons, Smart home, Health technology, Aging, Review

## Abstract

**Background:**

The worldwide increase in older persons demands technological solutions to combat the shortage of caregiving and to enable aging in place. Smart home health technologies (SHHTs) are promoted and implemented as a possible solution from an economic and practical perspective. However, ethical considerations are equally important and need to be investigated.

**Methods:**

We conducted a systematic review according to the PRISMA guidelines to investigate if and how ethical questions are discussed in the field of SHHTs in caregiving for older persons.

**Results:**

156 peer-reviewed articles published in English, German and French were retrieved and analyzed across 10 electronic databases. Using narrative analysis, 7 ethical categories were mapped: privacy, autonomy, responsibility, human vs. artificial interactions, trust, ageism and stigma, and other concerns.

**Conclusion:**

The findings of our systematic review show the (lack of) ethical consideration when it comes to the development and implementation of SHHTs for older persons. Our analysis is useful to promote careful ethical consideration when carrying out technology development, research and deployment to care for older persons.

**Registration:**

We registered our systematic review in the PROSPERO network under CRD42021248543.

**Supplementary Information:**

The online version contains supplementary material available at 10.1186/s12910-023-00898-w.

## Introduction/background

Significant advancements in medicine, public health and technology are allowing the world population to grow increasingly older adding to the steady rise in the proportion of senior citizens (aged over 65) [1]. Because of this growth in the aging population, the demand for and financial costs of caring for older adults are both rising [2]. That older persons generally wish to age in place and receive healthcare at home [2] may mean accepting risks such as falling, a risk that increases with frailty [3]. However, many prefer accepting these risks rather than moving into long term care facilities [4–6].

A solution to this multi-facetted problem of ageing safely at home and receiving appropriate care, while keeping costs at bay may be the use of smart home health technologies (SHHTs). A smart home is defined by Demiris and colleagues as “ residence wired with technology features that monitor the well-being and activities of their residents to improve overall quality of life, increase independence and prevent emergencies” [7]. SHHTs then, represent a certain type of smart home technology, which include non-invasive, unobtrusive, interoperable and possibly wearable technologies that use a concept called the Internet-of-Things (IoT) [8]. These technologies could thereby remotely monitor the older resident and register any abnormal deviations in the daily habits and vital signs while sending alerts to their formal and informal caregivers when necessary. These SHHTs could permit older people (and their caregivers) to receive the necessary medical support and attention at their convenience and will, thereby allowing them to continue living independently in their home environment.

All of these functions offer benefits to older persons wishing to age at home. While focusing on practical advantages is important, an equally important question to ask is how ethical these technologies are when used in the care of older persons. Principles of biomedical ethics, such as autonomy, justice [9], privacy [10], and responsibility [11] should not only be respected by medical professionals, but by technology developers and build-into the technologies as well.

The goal of our systematic review is therefore to investigate whether and which ethical concerns are discussed in the pertinent theoretical and empirical research on SHHTs for older persons between 2000 and 2020. Different from previous literature reviews [12–14],, which only explored practical aspects, we explicitly examined if and how researchers treated the ethical aspects of SHHTs in their studies, adding an important, yet often overlooked aspect to the systematic literature. Moreover, we present how and which ethical concerns are discussed in the theoretical literature and which ones in empirical literature, to shed light on possible gaps regarding which and how different ethical concerns are developed. Identifying these gaps is the first important step to eventually connecting bioethical considerations to the real world, adapting policies, guidelines and technologies itself [15]. Thus, our systematic review is the first one to do so in the context of ethical issues in SHHTs used for caregiving for older persons.

## Methods

### Search strategy

With the guidance of an information specialist from the University of Basel, our team developed a search strategy according to the PICO principle: Population 1 (Older adults), Population 2 (Caregivers), Intervention (Smart home health technologies), and Context (Home). The outcome of ethics was intentionally omitted as we wanted to capture all relevant studies without narrowing concerns that we would classify as “ethical”. Within each category, synonyms and spelling variations for the keywords were used to include all relevant studies. We then adapted the search string by using database-specific thesaurus terms in all ten searched electronic databases: EMBASE, Medline, PsycINFO, CINAHL, SocIndex, SCOPUS, IEEE, Web of Science, Philpapers, and Philosophers Index. We limited the search to peer-reviewed papers published between January 1st, 2000 and December 31st, 2020, written in the English, French, and German languages. This time frame allowed us to map the evolution to SHHTs as a new field.

The inclusion criteria were the following: (1) The article must be an empirical or theoretical original research contribution. Hence, book chapters, conference proceedings, newspaper articles, commentary, dissertations, and thesis were excluded. Also excluded were other systematic reviews since their inclusion would duplicate findings from our individual studies. (2) When the included study was empirical, the study’s population of interest must be older persons over 65 years of age, and/or professional or informal caregivers who provide care to older persons. Informal caregivers include anyone in the community who provided support without financial compensation. Professional caregivers include nurses and related professions who receive financial compensation for their caregiving services. (3) The included study must investigate SHHTs and their use in the older persons’ place of dwelling.

### Procedure

First, we carried out the systematic search across databases and removed all duplicates through EndNote (see supplementary Table 1 in appendix part 1 for a list of all included articles). One member of the research team screened all titles manually and excluded irrelevant papers. Then, two authors screened the abstracts and excluded irrelevant papers, and any disagreements were solved by a third author. She then also combined all included articles and removed further duplicates.

**Fig. 1:**
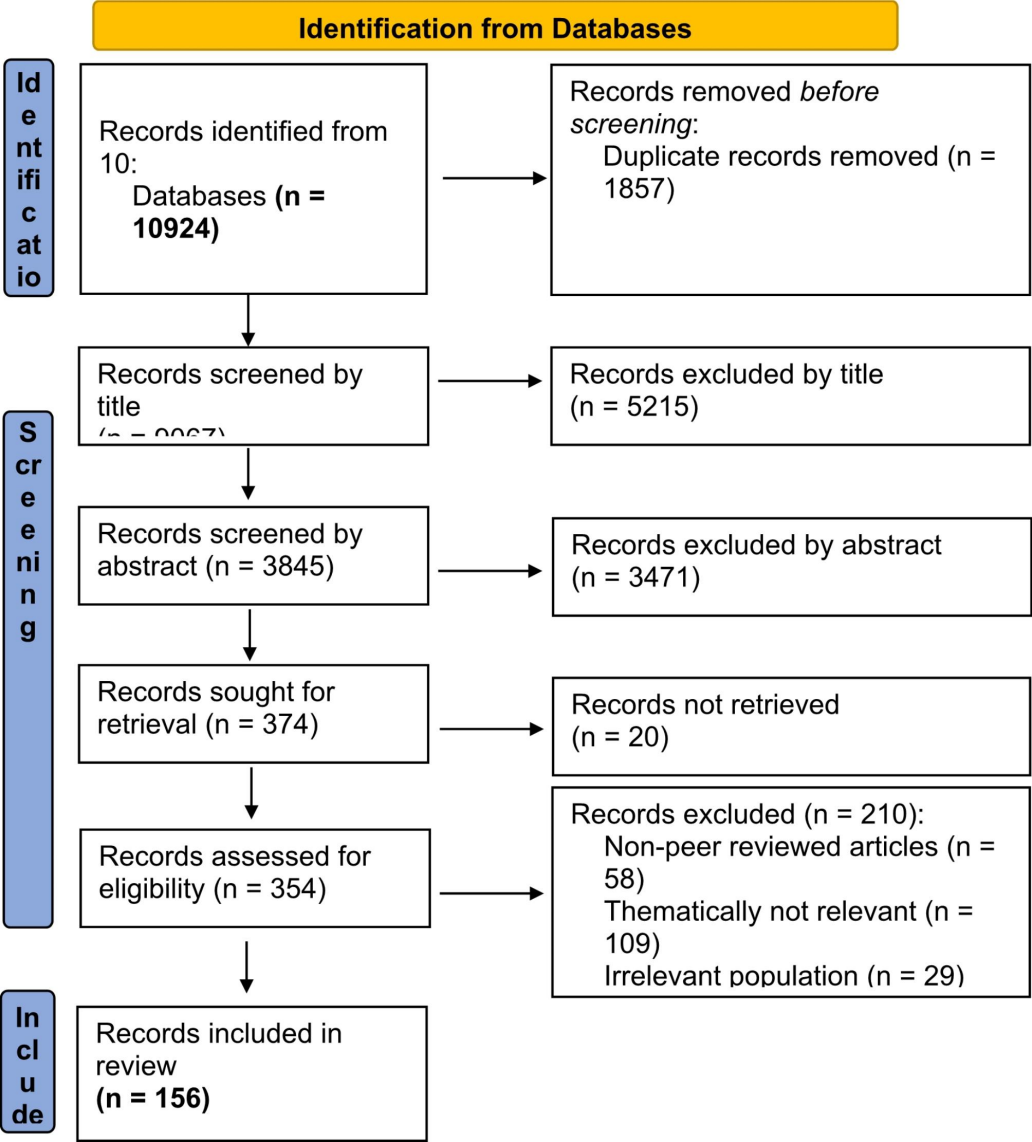
PRISMA 2020 Flowchart

### Final inclusion and data extraction

All included articles were searched and retrieved online (and excluded if full text was not available). Three co-authors then started data extraction, where several papers were excluded due to irrelevant content. To code the extracted data, a template was developed, which was tested in a first round of data extraction and then used in Microsoft Excel during the remaining extraction process. Study demographics and ethical considerations were recorded. Each extracting author was responsible for a portion of articles. If uncertainties or disputes occurred, they were solved by discussion. To ensure that our data extraction was not biased, 10% of the articles were reviewed independently. Upon comparing data extracted of those 10% of our overall sample, we found that items extracted reached 80% consistency.

### Data synthesis

The extracted datasets were combined and ethical discussions encountered in the publications were analyzed using narrative synthesis [16]. During this stage, the authors discussed the data and recognized seven first-order ethical categories. Information within these categories were further analyzed to form sub-categories that describe and/or add further information to the key ethical category.

## Results

### Nature of included articles

Our search initially identified 10,924 papers in ten databases. After the duplicates were removed, 9067 papers remained whose titles were screened resulting in exclusion of 5215 papers (Fig. 1). The examination of remaining 3845 abstracts of articles led to the inclusion of 374 papers for full-texts for retrieval. As we were unable to find 20 papers after several attempts, the remaining 354 full-texts were included for full-text review. In this full-text review phase, we further excluded 198 full-texts with reasons (such as technologies employed in hospitals, or technologies unrelated to health). Ultimately, this systematic review included 144 empirical and 12 theoretical papers specifying normative considerations of SHHTs in the context of caregiving for older persons.

Almost all publications (154 out of 156) were written in English, and over 67% [105] were published between 2014 and 2020. About a quarter (26%; 41 papers) were published between 2007 and 2013 and only 7% (10 articles) were from 2000 to 2006. Apart from the 12 theoretical papers, the methodology used in the 144 empirical papers included the following: 42 articles (29%) used a mixed-methods approach, 39 (27%) experimental, 38 (26%) qualitative, 15 (10%) quantitative, and the remaining were of an observational, ethnographical, case-study, or iterative testing nature.

The functions of SHHTs tested or studied in the included empirical papers were categorized as such: 29 articles (20.14%) were solely involved with (a) physiological and functioning monitoring technologies, 16 (11.11%) solely with (b) safety/security monitoring and assistance functions, 23 (15.97%) solely promoted (c) social interactions, and 9 (6.25%) solely for (d) cognitive and sensory assistance. However, 46 articles (29%) also involved technologies that fulfilled more than one of the categorized functions. The specific types of SHHTs included in this review comprised: intelligent homes (71 articles, 49.3%); assistive autonomous robots (49 articles, 34.03%); virtual/augmented/mixed reality (7, 4.4%); and AI-enabled health smart apps and wearables (4 articles, 1.39%). Likewise, the remaining 20 articles (12.8.8%) involved either multiple technologies or those that did not fall into any of the above categories.

### Ethical considerations

Of the 156 papers included, 55 did not mention any ethical considerations (See supplementary Table 1 in appendix part 1). Among the 101 papers that noted one or more ethical considerations, we grouped them into 7 main categories (1) privacy, (2) human vs. artificial relationships, (3) autonomy, (4) responsibility, (5) social stigma and ageism, (6) trust, and (7) other normative issues (see Table 1). Each of these categories consists of various sub-categories that provided more information on how smart home health technologies (possibly) affected or interacted with the older persons or caregivers in the context of caregiving (Table 2). Each of the seven ethical considerations are explained in depth in the following paragraphs.

**Table 1:** Ethically relevant topics mentioned in included manuscripts (N = 156)

Theme	# of empirical articles	# of theoretical articles	Total
Privacy	49	9	58
Human vs. Artificial Relationships	45	9	54
Autonomy	30	10	40
Responsibility	19	6	25
Ageism and Stigma	18	6	24
Trust	17	2	19
Other	0	4	4
None mentioned	55	0	55

**Table 2:** Specific concerns mentioned within each ethically relevant topic

Privacy	Human vs. artificial	Autonomy	Responsibility	Ageism and Stigma	Trust
General awareness	Importance of human caregiving	Control	Downsides of responsibility	Fear of being stigmatized by others	Characteristics promoting trust
Designing privacy	Fear of replacement of humans	Protecting autonomy/dignity	New responsibilities	Social Influence	General mistrust
Risk & Regulation	Preferences for technology	Importance of autonomy	Reducing burden of care	Exacerbating stigma for women	
Privacy in the case of cognitive impairment	Collaboration	Relational autonomy			

#### Privacy

This key category was cited across 58 articles. In theoretical articles, privacy was one of the most often discussed ethical consideration, as 9 out of 12 mentioned privacy related concerns. Among the 58 articles, four sub-issues within privacy were discussed.

(A)The awareness of privacy was reported as varying according to the type of SHHT end-user. Whereas some end-users were more aware or privacy in relation to SHHTs, others denoted little or a total lack of consideration, while some had differing levels of concerns for privacy that changed as it is weighed against other values, such as access to healthcare [17] or feeling of safety [18]. Both caregivers and researchers often took privacy concerns into account [19–21], while older persons themselves did not share the same degree of fears or concerns [22–24]. Older persons in fact were less concerned about privacy than costs and usability [23]. Furthermore, they were willing to trade privacy for safety and the ability to live at home. Nevertheless, several papers acknowledged that privacy is an individualized value, whereby its significance depends on both the person and their context, thus their preferences cannot be generalized [25–28]. Lastly, there were also some papers that explicitly stated that there were no privacy concerns found by the participants, or that participants found it useful to have monitoring without mentioning privacy as a barrier [29–31].

The second prevalent sub-issue within privacy was (B) privacy by choice. Both older persons and their caregivers expressed a preference for having a choice in technology used, in what data is collected, and where technology should or should not be to installed [32, 33]. For example, some spaces were perceived as more private and thus monitoring felt more intrusive [34–36]. Formal caregivers were concerned about monitoring technologies being used as a recording device for their work [37, 38]. Furthermore, older persons were often worried about cameras [39, 40] and “eyes watching”, even if no cameras were involved [41–43].

The third privacy concern was (C) risk and regulation of privacy, which included discussions surrounding dissemination of data or active data theft [44–47], as well as change in behavior or relationships due to interaction with technology [48, 49]. Researchers were aware of both legal and design-contextual measures that must be observed in order to ensure that these risks were minimized [45, 50, 51].

The final sub-issue that we categorized was (D) privacy in the case of cognitive impairment. This included disagreements if cognitive impairment warrants more intrusive measures or if privacy should be protected for everyone in the same way [52, 53].

#### Human versus artificial relationships

54 articles in our review contained data pertinent to trade-offs between human and artificial caregiving. Firstly, (A) there was a general fear that robots would replace humans in providing care for older persons [28, 54–56], along with related concerns such as losing jobs [40, 57], disadvantages with substituting real interpersonal contact [17, 46], and thus increasing the negative effects associated with social isolation [41, 58].

Many papers also emphasized (B) the importance of human caregiving, underlining the necessity of human touch [26, 47, 50, 59] believing that technology should and could not replace humans in connections [17], love [33], relationships [60], and care through attention to subtle signs of health decline in every in-person visit [57]. Older persons also preferred human contact over machines and had guarded reactions to purely virtual relationships[31, 61, 62]. The use of technology was seen to dehumanize care, as care should be inherently human-oriented [27, 48].

There was data alluding to (C) the positive reactions to technologies performing caregiving tasks and possibly forming attachments with the technology[47, 49, 58]. Furthermore, some papers cited participants reacting positively to robots replacing human care, where the concept of “good care” could be redefined [63–66]. Solely theoretical papers also identified possible benefits of tech for socialization and relationship building [67, 68].

Finally, many articles raised the idea of (D) collaboration between machine and human to provide caregiving to older persons [69]. These studies highlighted the possible harms if such collaboration was not achieved, such as informal caregivers withdrawing from care responsibilities [70] or the reinforcement of oppressive care relations [71]. Interestingly, opinions varied on whether the caregiving technology, such as a robot should have “life-like” appearance, voices, and emotional expressions, while recognizing the current technological limits in actually providing those features to a satisfactory level [46]. For example, some users preferred for the robot to communicate with voice commands, while others wanted to further customize this function with specific requests on the types of voices generated [65, 72].

#### Autonomy

40 papers mentioned autonomy of the older person with respect to the use of SHHTs. The first sub-theme categorized was in relation to (A) control, which encompassed positive aspects like (possible) empowerment through technology [25, 26, 73, 74] and negative aspects such as the possibility of technology taking control over the older person, thus increasing dependence [55, 75] or decreasing freedom of decision making [48]. Several studies reported the wishes of older persons to be in control when using the technology (e.g. technology should be easily switched off or on) and be in control of its potential, meaning the extend of data collected or transferred, for example [17, 30, 70, 76]. Furthermore, they should have the option to not use technology in spaces where they do not wish to, e.g., public spaces [35]. The issue of increased dependency was discussed as a loss or rather, fear of the loss of autonomy due to greater reliance on technology as well as the fear of being monitored all the time [28, 48]. In addition, using technology was deemed to make older persons more dependent and to increase isolation [77].

The second sub-category within autonomy highlighted the need for the technology to (B) protect the autonomy and dignity of its older end-users, which also included the unethical practice of deception (e.g.[46, 49, 54, 78], infantilization [31, 60], or paternalism [17, 27, 57], as a way to disrespect older persons’ dignity and autonomy [79–81]. Also reported was that these users may accept technology to avoid being a burden on others, thus underscoring the value of technology to enhance functional autonomy, understood here as independent functioning [52, 82, 83]. Other studies mentioned this kind of trade-off between autonomy and other values or interests as well. For example, between respecting the autonomy of the older persons versus nudging them towards certain behavior (perceived as beneficial for them) through the help of technology [32], or between autonomy and safety [24].

Two sub-issues within autonomy primarily discussed in the theoretical publications were (C) relational autonomy [27, 41, 49, 58] and (D) explanations on why autonomy should actually be preserved. The former emphasized the fact that older persons do not and should not live isolated lives and that there should be respect and promotion of their relationships with family members, friends, caregivers, and the community as a whole [27, 47]. The latter described the benefits of respecting autonomy, such as increased happiness and well-being [65, 67] or a sense of purpose [84], and thus favoring the promotion of autonomy and choice also from a normative perspective.

#### Responsibility

This theme included data across 25 articles that mentioned concerns such as the effect of using technologies on the current responsibilities of caregivers and older persons themselves. Specifically, the papers discussed (A) the downsides of assistive home technology on responsibility. That is, the use of technology conflicted with moral ideas around responsibility [58], especially for caregivers [57, 59]. Its use also raised more practical concerns, such as the fear of shifting the responsibility onto the technology and thus, diminishing vigilance and/or care. Related to this thought was also a fear of increased responsibility on both older persons [60] and their caregivers, who were worried about extra work time was needed to integrate technology into their work, learn its functions, analyze data, and respond to potentially higher frequencies of alerts [18, 35, 36, 53, 85].

Additionally, studies reported (B) continuous negotiation between (formal) caregivers’ (professional) responsibilities of care and the opportunities that smart technologies could provide [26, 47, 55, 70, 82]. For example, increased need for cooperation between informal and formal caregivers due to technology was foreseen [81] and fear expressed that over-reliance on female caregivers was exacerbated [71]. Nevertheless, the use of smart home health technologies was often seen to (C) reduce the burden of care, where caregivers could direct their attention and time to the most-needed situations and better align the responsibilities of care [5, 18, 49, 74, 80, 81]. This shift of burden onto a technology was also reported by older persons as freeing [48].

#### Ageism and stigma

24 articles discussed ageism and stigma, which included discussions about fear of (A) being stigmatized by others with the use of SHHTs [73, 86]. Older persons thought acceptance of such technologies also alluded to an admission of failure [82], or being perceived by others as frail, old, forgetful [77, 87], or even stupid [26, 33, 88]. This resulted in them expressing ageist views stating that they did not need the technology “yet” [84, 89]. Some papers reported the belief that the presence of robots was disrespectful for older people [52, 85, 90] and technologies do little to alleviate frustration and the impression of “being stupid” that older persons may have when they are faced with the complexities of the healthcare system [73]. Furthermore, older persons in a few studies did express unfamiliarity with learning new technologies in old age [42, 66, 91], coupled with fears of falling behind and not keeping up with their development, and feeling pressured to use technology [62, 89].

Within ageism and stigma, (B) social influence was deemed to cause older persons to believe that the longer they have been using technology, the more their loved ones want them to use it as well, creating a sort of reinforcing loop [27]. Other social points were related to self-esteem, meaning that older persons needed to reach a certain threshold first to publicly admit that they need technology [85], or doubts by caregivers if they were able to use the devices [36]. This possibly led older persons to prefer unobtrusive technology and those that could not be noticed by visitors [22, 55, 88].

Lastly, (C) two theoretical articles raised concerns in regard to technology exacerbating stigmatization of women and migrants in caregiving. Both Parks [47] and Roberts & Mort [71] suggested that caregiving technology which does not question the underlying expectation that women give care to their relatives will worsen such gendered expectations in caregiving.

#### Trust

We identified 18 articles that mentioned some aspect of trust. For both older persons and caregivers, there was often (A) a general mistrust with technologies compared with existing human caregiving [33, 42]. Therefore, caregivers became proxies and were relied on to “understand it” and continue providing care [48]. For caregivers the lack of trust was associated with the use of technologies, for example, leaving older persons alone with technology [81], worrying that older persons would not trust the technology [29, 32] or that it could change their professional role [23]. One paper even reported that using technology meant caregivers themselves are not trusted [92]. Surprisingly, some studies found that older persons had no problem trusting technology, even considering it safer and more reliable than humans [58, 70].

The second sub-theme concerned (B) characteristics promoting trust. That is, the degree of automation [30](, the involvement of trusted humans in design and use [34, 93], perceived usefulness of the technology and spent time with the technology all influenced trust [59, 72, 94]. For robots specifically, they were trusted more than virtual agents, such as Alexa [60, 65]. Taking this step further, studies discovered that robots with a higher degree of automation or a lower degree in anthropomorphism level increased trust [30].

#### Other

There were several miscellaneous considerations not fitting the ones already mentioned above, and we categorized them as follows. Firstly, two theoretical articles mentioned (A) considerations related to research. Ho, [27] pointed out that empirical evidence of the usefulness of SHHTs is lacking, which therefore may make them less relevant as a possible solution for aging in place. Palm et al. (2013) suggested that, if research would consider the fact that many costs of caregiving are hidden because of non-paid informal caregivers, the actual economic benefits of SHHTs are unknown. Lastly, two articles alluded to (B) psychological phenomena related to the use of SHHTs. Pirhonen et al., [58] suggested that robots can promote the ethical value of well-being through the promotion of feelings of hope. The other phenomenon was feeling of blame and fear associated with the adoption of the technology, as caregivers may be pushed to use SHHTs in order to not be blamed for failing to use technology [18]. This then also nudged caregivers to think that using SHHTs cannot do any harm, so it is better to use it than not use it.

## Discussion

Our systematic review investigated if and how ethical considerations appear in the current research on SHHTs in the context of caregiving for older persons. As we included both empirical and theoretical works of literature, our review is more comprehensive that existing systematic reviews (e.g.[12–14], that have either only explored the empirical side of the research and neglected to study ethical concerns. Our review offers an informative and useful insights on dominant ethical issues related to caregiving, such as autonomy and trust [95, 96]. At the same time, the study findings brings forth less known ethical concerns that arise when using technologies in the caregiving context, such as responsibility [97] and ageism and stigma.

The first key finding of our systematic review is the silence on ethics in SHHTs research for caregiving purposes. Over a third of the reviewed publications did not mention any ethical concern. One possible explanation is related to scarcity [98]. In the context of research in caregiving for older persons, “scarcity” can be understood in a variety of ways: one way is to see the available space for ethical principles in medical technology research as scarce. For example, according to Einav & Ranzani [99] “Medical technology itself is not required to be ethical; the ethics of medical technology revolves around when, how and on whom each technology is used” (p.1612). Determining the answers to these questions is done empirically, by providing proof of benefit of the technology, ongoing reporting on (possibly harmful) long term effects, and so on [99]. Given that publication space in journal is limited to a certain amount of text, the available space that ethical considerations can take up is scarce. Therefore, adding deliberations about the unearthed values or issues in our systematic review, like trust, responsibility or ageism, may simply not fit in the space available in research publications. This may also be the reason why the values of beneficence and non-maleficence were not found through our narrative analysis. While both values are considered crucial in biomedical ethics [9], the empirically measured benefits may be considered enough by the authors to demonstrate beneficence (and non-maleficence), leading them to not mention the ethical values explicitly again in their publications.

Another interpretation is the scarcity of time, and the felt pressure to “solve” the problem of limited resources in caregiving [2]. Researchers might be therefore more inclined to focus on the empirical data showing benefits, rather than to engage in elaborations on ethical issues that arise with those benefits. Lastly, as researchers have to compete for limited funding [100] and given that technological research receives more funding than biomedical ethics [101], it is likely that the numbers of publications mentioning purely empirical studies exceeds those publications that solely mention the ethical issues (as our theoretical papers did) or that combine empirical and ethical parts. Further research needs to investigate these hypotheses further.

It is not surprising that privacy was the most discussed ethical issue in relation to SHHTs in caregiving. The topic of privacy, especially in relation to monitoring technologies and/or health, has been widely discussed (see for example [102–104]. A particularly interesting finding within this ethical concern was related to privacy and cognitive impairment. While discussions around autonomy and cognitive impairment are popular in bioethical research (see e.g. [105, 106], privacy, on the other hand, has recently gained more attention for both researchers and designers [107]. The relation in the reviewed studies between cognitive impairment and privacy seemed to be reversely correlated –intrusions into the privacy of older persons with cognitive impairments were deemed as more justified [35, 53], which necessarily does not mean that its ethical, but a practical fact that such intrusions become possible or necessary in the given context. A possible explanation lies in the connectedness of autonomy and privacy, in the sense that autonomy is needed to consent for any sort of intrusions [108].

Surprisingly, more research papers mentioned the topic of human vs. artificial relationships as an ethical concern than autonomy. Autonomy is often the most discussed ethics topic when it comes to use of technology [96]. However, fears associated with technology replacing human care has recently gained traction [109–111].The significance of this theme is likely due to the fact that caregiving for older persons has been (and is) a very human-centric activity [112]. As mentioned before, the persons willing and able to do this labor (both paid and unpaid caregiver) are limited and their pool is shrinking [113]. The idea of technology possibly filling this gap is not new [114], but is also clearly causing wariness among both older persons and caregivers, as we have discovered [56, 61]. Frequently mentioned was the fear of care being replaced by technology. This finding was to be expected, as nursing is not the only profession where introduction of technology caused fears of job loss [115]. Within this ethical concern, the importance of human touch and human interaction was underlined [110, 111]. Human touch is an important asset for caregivers when they care for older patients, particularly those with dementia, as it is one of the few ways to establish connection and to calm the patient with dementia [116]. Similarly, human touch and face-to-face interactions are mentioned as a critical aspect of caregiving in general, both for the care recipient and the caregiver [117, 118]. While caregivers see the aspect of touching and interacting with older care recipients as a way to make their actions more meaningful and healing [90, 117], for care recipients being touched, talked and listened to is part of feeling respected and experiencing dignity [118, 119]. Introducing technology into the caregiving profession may therefore quickly elicit associations with cold and lifeless objects [59]. Future developments, both in the design of the technologies themselves and their implementation in caregiving will require critical discussion among concerned stakeholders and careful decision on how and to what extent the human touch and human care must be preserved.

A unique ethical concern that we have not seen in previous research [120, 121] is responsibility, and remarkable within this concern was SHHTs’ negative impact on it. As previously mentioned, the human being and human interaction are seen as central to caregiving [117, 118]. This can possibly be extended to concepts exclusively attributable to humans, such as the concept of moral responsibility [122]. Shifting caregiving tasks onto a technological device, which, by being a device and not a human carer, cannot be morally responsible in the same way as a human being can [123], may introduce a sense of void that caregivers are reluctant to create. Studies have shown that a mismatch in professional and personal values in nursing causes emotional discomfort and stress [124], therefore the shift in the professional environment caused by SHHTs is likely to be met with aversion. Additionally, the negative impact of SHHTs on caregiving responsibility was also tied to practical concerns, like not having enough time to learn how to use the technology by the caregivers [35], or needing to have access to and checking the older person’s health data [36]. Such concerns point to the possibility that SHHTs can create unforeseen tasks, which could turn into true burdens, instead of alleviating caregivers. Indeed, there are indications that the increase in information about the older person through monitoring technologies causes stress for both caregivers and older persons, as the former feel pressure to look at the available data, while the latter prefer to hide unfavorable information to not seem burdensome for their caregivers [125]. Another consequence of SHHTs that emerged as a sub-category was the renegotiation of responsibilities among the different stakeholders. In the field of (assistive) technology, this renegotiation is an ongoing process with efforts to make technology and its developers more accountable, through new policies and regulations [126]. In the realm of assistive technology in healthcare, these negotiations focus on high-risk cases and emergencies [127]. Who is responsible for the death of a person if the assistive technology failed to recognize an emergency, or to alert humans in time? Such issues around responsibility and legal liability are partially responsible for the slow uptake of technology in caregiving [128].

Another important but less discussed ethical concern was ageism and stigma. Ageist prejudices include being perceived as slow, useless, burdensome, and incompetent [129]. Fear of aging and becoming a burden to others is a fear many older persons have, as current social norms demand independence until death [130]. Furthermore, the general ubiquitous use of technology has possibly exacerbated the issue of ageism, as life became fast paced and more pressure is placed on aging persons to keep up [131]. While this would call for more attention to studying ageism in relation to technology, our findings indicate that, it does not unfortunately seem at the forefront of concerns that are prevalent in the literature (and thereby the society).

Related to ageism, is the wish of older persons to not be perceived as old and/or in the need of assistance (in the form of technology) explains the prevalent demand for unobtrusive technology. Obtrusiveness, in the context of SHHTs, is defined as “undesirably prominent and or/noticeable”, yet this definition should include the user’s perception and environment, and is thus not an objectively applicable definition [132]. Nevertheless, we can infer that by “unobtrusive”, users mean SHHTs that is not noticeable by them or, mostly importantly, by other persons to possibly reduce stigma associated with using a technology deemed to be for persons with certain limitations. Further research will have to confirm if unobtrusive technology actually reduces stigma and/or fosters acceptance of such SHHTs in caregiving.

Lastly, the sub-theme of stigmatization of women and immigrants in caregiving and possibly exacerbating their caregiving burden through technology was only discovered in two theoretical publications [47, 71]. While it is well known that caregiving burden mostly falls upon women [133, 134], many of them with a migration background when it comes to live-in caregivers [135, 136]. It is surprising that we found no redistribution of burden of care with technology. This is likely due to the fact that caregiving – be it technologically assisted or not – remains perceived as a more feminine and, unfortunately, low status profession [137]. The development of technology, however, are still mostly associated with masculinity This tension between the innovators and actual users of technology can lead to the exacerbation of stigma for female and migrant caregivers, as the human bias is conserved by the technology, instead of disrupted through it [137].

Finally, trust was an expected ethical concern, given that it is a widely discussed topic in relation to technology (see for example, [123, 138] and also in the context of nursing [95, 139]. Older persons were trusting caregivers to understand SHHTs [48], while caregivers feared that older persons would not trust the used technology, even though said persons did not express such concerns [32]. A possibility to mitigate such misunderstandings and put both caregivers and care recipients on an equal understanding of the technology are education tools [140]. Another surprising finding was that some older persons were inclined to trust SHHTs even more than human caregivers, as they were seen as more reliable [70]. This trust in technology was increased when a physical robot instead of an only virtual agent was involved [60, 65]. Studies in the realm of embodiment of virtual agents and robots suggest that the presence of a body or face promotes human-like interactions with said agents [51]. Furthermore, our systematic review discovered other characteristics which promote trust in SHHTs, such as perceived usefulness [94] or time spent with the technology [59]. Another important aspect is the already existing trust in the person introducing the technology to the user [34, 93]. In combining these characteristics in the design and implementation of SHHTs in caregiving, researchers and technology developers need to find creative mechanisms to facilitate trustworthiness and foster adoption of new technologies in caregiving.

## Limitations

While we searched 10 databases for publications over a span of 20 years, we are aware that older or newer publications will have escaped our systematic review. Relevant new literature that we have found when writing our results have been incorporated in this manuscript. Furthermore, as we specifically refrained from using terms related to ethics in our search strings to also capture the instances of absence of ethical concerns, this choice may have led to missing a few articles as a consequence, especially in regards to theoretical publications. Lastly, due to lack of resources, we were unable to carry out independent data extraction for all included papers (N = 156) and chose to validate the quality of extracted data by using a random selection of 10% of the included sample. Since there was high agreement on extracted data, we are confident about the quality of our study findings.

## Conclusion

SHHTs offer the possibility to mitigate the shortage of human caregiving resources and to enable older persons to age in place, being adequately supported by technology. However, this shift in caregiving comes with ethical challenges. If and how these ethical challenges are mentioned in the current research around SHHTs in caregiving for older persons was the goal of this systematic review. Through analyzing 156 articles, both empirical and theoretical, we discovered that, while over one third of articles did not mention any ethical concerns whatsoever, the other two thirds discussed a plethora of ethical issues. Specifically, we discovered the emergence of concerns with the use of technology in the care of older persons around the theme of human vs. artificial relationships, ageism and stigma, and responsibility. In short, our systematic review offers a comprehensive overview of the currently discussed ethical issues in the context of SHHTs in caregiving for older persons. However, scholars in the fields of gerontology, ethics, and technology working on such issues would be already (or should be) aware that ethical concerns will change with each developing technology and the population it is used for. For instance, with the rise of Artificial intelligence/Machine Learning, new intelligent or smart technologies will continue to mature with use and time. Thus, ethical value such as autonomy will require re-evaluation with this significant content development as well as deciding, if the person would/should be asked to re-consent or how should this decision making proceed should he or she have developed dementia. In sum, more critical work is necessary to prospectively act on ethical concerns that may arise with new and developing technologies that could be used in reducing caregiving burden now and in the future.

## Electronic supplementary material

Below is the link to the electronic supplementary material.

Additional File 1: PRISMA 2020 checklist

Additional File 2: Appendix part 1

Additional File 3: Appendix part 2

## Data Availability

All data generated or analyzed during this systematic review are included in this published article and its appendices. Appendix part 1 contains all included articles and their characteristics. Appendix part 2 contains the search strategy and all search strings for all searched databases, as well as the PROSPERO registration number.
