# Supplementary material for: Mapping ethical issues in the use of smart home health technologies to care for older persons: a systematic review
Source: BMC Med Ethics. 2023 Mar 29;24:24. doi: 10.1186/s12910-023-00898-w (PMC10061702; doi:10.1186/s12910-023-00898-w)
Supplement: Supplementary file 2 — Additional File 2: Appendix part 1 [file 12910_2023_898_MOESM2_ESM.docx]

**Appendix Part 1**

**Supplementary Table 1** – Included Articles, their Characteristics and Type of Ethical Concerns mentioned

| # | Year of publication | Last Name first author | Title | Type of paper | Ethical concerns mentioned |
| --- | --- | --- | --- | --- | --- |
| 1 | **2005** | **Kelly** | Smart support at home: the integration of telecare technology with primary and community care systems | Theoretical | Autonomy, human vs. artificial |
| 2 | **2009** | **Demiris** | "Smart homes" for patients at the end of life | Theoretical | Privacy, autonomy, responsibility, human vs. artificial, stigma, other |
| 3 | **2009** | **Roberts** | Reshaping what counts as care: Older people, work and new technologies | Theoretical | Responsibility, human vs. artificial, stigma, |
| 4 | **2010** | **Kang** | In situ monitoring of health in older adults: Technologies and issues | Theoretical | Privacy, autonomy, human vs. artificial, stigma, practical |
| 5 | **2013** | **Palm** | Who cares? Moral obligations in formal and informal care provision in the light of ICT-based home care | Theoretical | Autonomy, response, trust, human vs. artificial, other |
| 6 | **2014** | **Mitzner** | Identifying the Potential for Robotics to Assist Older Adults in Different Living Environments | Theoretical | Autonomy, human vs. artificial |
| 7 | **2015** | **Park** | Home-Based Care, Technology, and the Maintenance of Selves | Theoretical | Privacy, autonomy, responsibility, human vs. artificia, stigma |
| 8 | **2017** | **Preuss** | Living with the animals: animal or robotic companions for the elderly in smart homes? | Theoretical | Privacy, autonomy, responsibility, human vs. artificia, stigma |
| 9 | **2019** | **Li** | Smart homes for healthcare | Theoretical | Privacy |
| 10 | **2019** | **Moyle** | The promise of technology in the future of dementia care | Theoretical | Privacy, autonomy, human vs. artificia, stigma, others |
| 11 | **2020** | **Ho** | Are we ready for artificial intelligence health monitoring in elder care? | Theoretical | Privacy, autonomy, responsibility, stigma, practical |
| 12 | **2020** | **Pirhonen** | ould robots strengthen the sense of autonomy of older people residing in assisted living facilities?—A future-oriented study | Theoretical | Privacy, autonomy, trust, responsibility, human vs. artificial, other |
| 13 | **2000** | **Sixsmith** | An evaluation of an intelligent home monitoring system | Mixed Methods | Privacy, human vs. artificia, |
| 14 | **2002** | **Ohta** | A health monitoring system for elderly people living alone | Observational | None |
| 15 | **2003** | **Pineau** | Towards robotic assistants in nursing homes: Challenges and results | Experimental | None |
| 16 | **2004** | **Graf** | Care-o-bot II - Development of a next generation robotic home assistant | Quantitative | None |
| 17 | **2004** | **Kinney** | Striving to Provide Safety Assistance for Families of Elders:The SAFE House Project | Qualitative | Privacy, responsibility |
| 18 | **2004** | **Libin** | Therapeutic robocat for nursing home residents with dementia: Preliminary inquiry | Experimental | Human vs. artificial |
| 19 | **2004** | **Tamura** | Is an Entertainment Robot Useful in the Care of Elderly People with Severe Dementia? | Experimental | Human vs. artificial |
| 20 | **2005** | **Morris** | Social networks as health feedback displays | Qualitative | Autonomy, responsibility |
| 21 | **2006** | **Bharucha** | Ethical considerations in the conduct of electronic surveillance research | Qualitative | Privacy |
| 22 | **2007** | **Bertera** | A study of the receptivity to telecare technology in a community-based elderly minority population | Quantitative | Privacy |
| 23 | **2007** | **Boissy** | A qualitative study of in-home robotic telepresence for home care of  community-living elderly subjects | Qualitative | Privacy, autonomy, human vs. artificial |
| 24 | **2007** | **Reeves** | A trial of telecare for supporting care to the elderly in Liverpool | Experimental | None |
| 25 | **2008** | **Alexander** | Sensor systems for monitoring functional status in assisted living facility residents | Experimental | None |
| 26 | **2008** | **Banks** | Animal-assisted therapy and loneliness in nursing homes: use of robotic versus living dogs | Mixed methods | Privacy, human vs. artificial |
| 27 | **2008** | **Essén** | The two facets of electronic care surveillance: An exploration of the views of older people who live with monitoring devices | Qualitative | Privacy, autonomy, responsibility, trust, human vs. artificial |
| 28 | **2008** | **Mazzu** | Wireless-accessible sensor populations for monitoring biological variables | Quantitative | None |
| 29 | **2008** | **Wai** | Smart wireless continence management system for persons with dementia | Observational | None |
| 30 | **2009** | **Fauconau** | Caregivers' requirements for in-home robotic agent for supporting community-living elderly subjects with cognitive impairment | quantiative | Privacy, Ageism |
| 31 | **2009** | **Kramer** | Comparison of the Effect of Human Interaction, Animal-Assisted Therapy, and AIBO-Assisted Therapy on Long-Term Care Residents with Dementia | Mixed Methods | None |
| 32 | **2009** | **Londei** | An intelligent videomonitoring system for fall detection at home: perceptions of elderly people | Mixed Methods | Privacy, autonomy, ageism |
| 33 | **2010** | **Govercin** | Defining the user requirements for wearable and optical fall prediction and fall detection devices for home use | Mixed Methods | Privacy |
| 34 | **2010** | **Junnila** | Wireless, Multipurpose In-Home Health Monitoring  Platform: Two Case Trials | Experimental | None |
| 35 | **2010** | **Rowe** | Sleep in Dementia Caregivers and the Effect of a Nighttime Monitoring System | Mixed Methods | None |
| 36 | **2011** | **Alexander** | Evolution of an Early Illness Warning System toMonitor Frail Elders in Independent Living | Mixed Methods | None |
| 37 | **2011** | **Alexander** | Passive Sensor Technology Interface to Assess Elder Activity in Independent Living | Experimental | None |
| 38 | **2011** | **Mahoney** | An evidence-based adoption of technology model for remote monitoring of elders' daily activities | Mixed Methods | Privacy, human vs. artificial |
| 39 | **2011** | **Miligan** | Telecare and older people: Who cares where? | Qualitative | Privacy, autonomy, human vs. artificial |
| 40 | **2011** | **Van Berlo** | Experiences with smart homes for older people | Observational | Autonomy |
| 41 | **2012** | **Hossain** | Virtual Caregiver: An Ambient-Aware Elderly Monitoring System | Mixed Methods | None |
| 42 | **2012** | **Mitseva** | Gerontechnology: Providing a helping hand when caring for cognitively impaired older adults-intermediate results from a controlled study on the satisfaction and acceptance of informal caregivers | Experimental | Ageism |
| 43 | **2012** | **Patel** | A passive monitoring system in assisted living facilities: 12-month comparative study | Experimental | None |
| 44 | **2012** | **Seelye** | Reactions to a remote-controlled video-communication robot in seniors' homes: A pilot study of feasibility and acceptance | Experimental | Privacy |
| 45 | **2012** | **Suryadevara** | Wireless Sensor Network Based Home Monitoring System for Wellness Determination of Elderly | Experimental | None |
| 46 | **2012** | **Tanaka** | Effect of a human-type communication robot on cognitive function in elderly women living alone | Experimental | None |
| 47 | **2013** | **Aloulou** | Deployment of assistive living technology in a nursing home environment: methods and lessons learned | Mixed methods | Privacy, |
| 48 | **2013** | **Bayen** | Evaluating the effectiveness of a memory aid system | Experimental | None |
| 49 | **2013** | **Boise** | Willingness of older adults to share data and privacy concerns after exposure to unobtrusive in-home monitoring | Quantitative | Privacy |
| 50 | **2013** | **Bowes** | Telecare for Older People: Promoting Independence, Participation, and Identity | Qualitative | Autonomy, ageism |
| 51 | **2013** | **Frennert** | Elderly People's Perceptions of a Telehealthcare System: Relative Advantage, Compatibility, Complexity and Observability | Qualitative | Privacy, autonomy, human vs. artificial, ageism |
| 52 | **2013** | **Marti** | Exploring empathy in interaction | Qualitative | Autonomy, trust, human vs. artificial |
| 53 | **2013** | **Matsui** | Development of a practicable non-contact bedside autonomic activation monitoring system using microwave radars and its clinical application in elderly people | Experimental | None |
| 54 | **2013** | **Melkas** | Innovative assistive technology in Finnish public elderly-care services: A focus on productivity | Mixed methods | Ageism |
| 55 | **2013** | **Peter** | AGNES: Connecting people in a multimodal way | Mixed methods | Privacy, ageism |
| 56 | **2013** | **Robert** | SWEET-HOME ICT technologies for the assessment of elderly subjects | Quantitative | None |
| 57 | **2013** | **Tseng** | Designing an intelligent health monitoring system and exploring user acceptance for the elderly | Experimental | None |
| 58 | **2013** | **Zsiga** | Home care robot for socially supporting the elderly: Focus group studies in three European countries to screen user attitudes and requirements | Qualitative | Privacy, autonomy, human vs. artificial |
| 59 | **2014** | **Alexander** | Generating Sensor Data Summaries to Communicate Change in Elder's Health Status | Experimental | None |
| 60 | **2014** | **Meiland** | Participation of end users in the design of assistive technology for people with mild to severe cognitive problems; the European Rosetta project | Qualitative | Privacy, human vs. artificial, other |
| 61 | **2014** | **Torta** | Evaluation of a Small Socially-Assistive Humanoid Robot in Intelligent Homes for the Care of the Elderly | Experimental | Trust, human vs. artificial |
| 62 | **2015** | **Anderson** | The Impact of Assistive Technologies on Formal  and Informal Home Care | Quantitative | None |
| 63 | **2015** | **Cai** | Health professionals' user experience of the intelligent bed in patients' homes | Mixed methods | Privacy |
| 64 | **2015** | **Canally** | Using integrated bio-physiotherapy informatics in home health-care settings: A qualitative analysis of a point-of-care decision support system | Mixed methods | None |
| 65 | **2015** | **Cavallo** | An ambient assisted living approach in designing domiciliary services combined with innovative technologies for patients with Alzheimer's disease: a case study | Mixed methods | Privacy, autonomy, trust |
| 66 | **2015** | **Jenkins** | Care, Monitoring, and Companionship: Views on Care Robots from Older People and Their Carers | qualitative | Privacy, autonomy, responsibility, trust, human vs. artificial |
| 67 | **2015** | **Lamprinakos** | An integrated remote monitoring platform towards Telehealth and Telecare services interoperability | quantitative | Privacy |
| 68 | **2015** | **Lee** | Sensor-based observations of daily living for aging in place | qualitative | None |
| 69 | **2015** | **Pérez** | Caregiver and social assistant robot for rehabilitation and coaching for the elderly | quantitative | None |
| 70 | **2015** | **Pfadenhauer** | Robot Caregiver or Robot-Supported Caregiving?: The Performative Deployment of the Social Robot PARO in Dementia Care | Ethnography | Responsibility, human vs. artificial |
| 71 | **2015** | **Rose** | Correlates Among Nocturnal Agitation, Sleep, and Urinary Incontinence in Dementia | Mixed methods | Responsibility |
| 72 | **2015** | **Sung** | Robot-assisted therapy for improving social interactions and activity participation among institutionalized older adults: A pilot study | Experimental | None |
| 73 | **2015** | **Valenti** | Social robots in advanced dementia | Experimental | None |
| 74 | **2016** | **Birks** | Robotic Seals as Therapeutic Tools in an Aged Care Facility: A Qualitative Study | Qualitative | Privacy, human vs. artificial, other |
| 75 | **2016** | **Chaumon** | Detecting falls at home: User-centered design of a pervasive technology | Mixed methods | Privacy, autonomy, responsibility, human vs. artificial, ageism |
| 76 | **2016** | **Cohen** | Acceptability of an intelligent wireless sensor system for the rapid detection of health issues: findings among home-dwelling older adults and their informal caregivers | Mixed methods | Privacy |
| 77 | **2016** | **Eldib** | Behavior analysis for elderly care using a network of low-resolution visual sensors | Experimental | None |
| 78 | **2016** | **Epstein** | Older Adults' and Caregivers' Perspectives on In-Home Monitoring Technology | Mixed methods | Privacy, trust, human vs. artificial, ageism |
| 79 | **2016** | **Iio** | Social acceptance by senior citizens and caregivers of a fall detection system using range sensors in a nursing home | Qualitative | Privacy |
| 80 | **2016** | **Jøranson** | Change in quality of life in older people with dementia participating in Paro-activity: a cluster-randomized controlled trial | Experimental | None |
| 81 | **2016** | **Lazarou** | A Novel and Intelligent Home Monitoring System for Care Support of Elders with Cognitive Impairment | Mixed methods | None |
| 82 | **2016** | **Saunders** | 'Teach Me-Show Me'-End-User Personalization of a Smart Home and Companion Robot | Mixed methods | None |
| 83 | **2017** | **Baisch** | Emotionale Roboter im  Pflegekontext  Empirische Analyse des bisherigen Einsatzes  und derWirkungen von Paro und Pleo | Mixed methods | Human vs. artificial, other |
| 84 | **2017** | **Chung** | Feasibility testing of a home-based sensor system to monitor mobility and daily activities in Korean American older adults | Mixed methods | Privacy, trust, |
| 85 | **2017** | **Draper** | Ethical Values and Social Care Robots for Older People: An International Qualitative Study | qualitative | Privacy, autonomy, human vs. artificial |
| 86 | **2017** | **Dupuy** | Everyday functioning benefits from an assisted living platform amongst frail older adults and their caregivers | experimental | None |
| 87 | **2017** | **Görer** | An autonomous robotic exercise tutor for elderly people | Mixed methods | Responsibility, trust, human vs. artificial, other |
| 88 | **2017** | **Hall** | Implementing monitoring technologies in care homes for people with dementia: A qualitative exploration using Normalization Process Theory | Qualitative | Privacy, responsibility, other |
| 89 | **2017** | **Jung** | An Exploration of the Benefits of an animallike Robot Companion with more advanced touch interaction capabilities for Dementia Care | Quantitative | Human vs. artificial |
| 90 | **2017** | **Kim** | Unobtrusive Monitoring to Detect Depression for Elderly With Chronic Illnesses | Mixed methods | Privacy |
| 91 | **2017** | **Klemets** | Nurses' Perspectives on In-Home Monitoring of Elderlies's Motion Pattern | qualitative | None |
| 92 | **2017** | **Korchut** | Challenges for service robots-requirements of elderly adults with cognitive impairments | Mixed methods | Autonomy, responsibility, human vs. artificial, ageism |
| 93 | **2017** | **Liang** | A Pilot Randomized Trial of a Companion Robot for People With Dementia Living in the Community | Experimental | Autonomy, human vs. artificial, |
| 94 | **2017** | **Moyle** | Use of a Robotic Seal as a Therapeutic Tool to Improve Dementia Symptoms: A Cluster-Randomized Controlled Trial | Experimental | None |
| 95 | **2017** | **Naick** | Innovative approaches of using assistive technology to support carers to care for people with night-time incontinence issues | Case study | Human vs. artificial |
| 96 | **2017** | **Rantanen** | An In-home Advanced Robotic System to Manage Elderly Home-care Patients’ Medications: A Pilot Safety and Usability Study | Mixed methods | Human vs. artificial |
| 97 | **2017** | **Rantz** | Randomized Trial of Intelligent Sensor System for Early Illness Alerts in Senior Housing | Experimental | None |
| 98 | **2017** | **Sanchez** | ICT Services for Life Improvement for the Elderly | Iterative testing | None |
| 99 | **2017** | **Urwyler** | Cognitive impairment categorized in community-dwelling older adults with and without dementia using in-home sensors that recognise activities of daily living | Empirical | Privacy |
| 100 | **2017** | **Vanderberg** | US and Dutch nurse experiences with fall prevention technology within nursing home environment and workflow: A qualitative study | Empirical | None |
| 101 | **2017** | **Wang** | Robots to assist daily activities: Views of older adults with Alzheimer's disease and their caregivers | Empirical | Human vs. artificial, ageism |
| 102 | **2018** | **Bakas** | Satisfaction and Technology Evaluation of a Telehealth Robotic Program to Optimize Healthy Independent Living for Older Adults | Empirical | None |
| 103 | **2018** | **Barnier** | Building Automation, an Acceptable Solution to Dependence? Responses Through an Acceptability Survey About a Sensors Platform | Empirical | Privacy, human vs. artificial |
| 104 | **2018** | **Bradford** | Watching over me: positive, negativeand neutral perceptions of in-homemonitoring held by independent-livingolder residents in an Australian pilot study | Empirical | Privacy, autonomy, trust, ageism |
| 105 | **2018** | **Gokalp** | Integrated Telehealth and Telecare for Monitoring Frail Elderly with Chronic Disease | Empirical | Ageism |
| 106 | **2018** | **Klein** | A robotic shower system: Acceptance and ethical issues | Empirical | Privacy, responsibility, human vs. artificial |
| 107 | **2018** | **Sadek** | Nonintrusive Remote Monitoring of Sleep in Home-Based Situation | Empirical | None |
| 108 | **2018** | **Wilkinson** | Monitoring Health Status in Long Term Care Through the Use of Ambient Technologies and Serious Games | Empirical | None |
| 109 | **2018** | **Wright** | Tactile care, mechanical Hugs: Japanese caregivers and robotic lifting devices | Empirical | Human vs. artificial, ageism |
| 110 | **2019** | **Annica** | Summative evaluation of a sensor-based cognitive  assistive technology: Impact on quality of life and  perceived utility | Empirical | None |
| 111 | **2019** | **Barrett** | Evaluation of a Companion Robot for Individuals with Dementia - Quantitative findings of the MARIO Project in an Irish Residential Care Setting | Empirical | Privacy |
| 112 | **2019** | **Bedaf** | What are the preferred characteristics of a service robot for the elderly? A multi-country focus group study with older adults and caregivers | Empirical | Privacy, autonomy, trust |
| 113 | **2019** | **Berridge** | Sensor-Based Passive Remote Monitoring and Discordant Values:Qualitative Study of the Experiences of Low-Income ImmigrantElders in the United States | Empirical | Autonomy (deception), human vs. artifical |
| 114 | **2019** | **Cahill** | IoT/Sensor-Based Infrastructures Promoting a Senseof Home, Independent Living, Comfort and Wellness | Empirical | Privacy, autonomy |
| 115 | **2019** | **De la Puente** | Grasping Objects From the Floor in Assistive Robotics: Real World Implications and Lessons Learned | Empirical | None |
| 116 | **2019** | **Delmastro** | Experimenting mobile and e-health services with frail MCI older people | Empirical | None |
| 117 | **2019** | **Erebak** | Caregivers’ Attitudes Toward Potential Robot Coworkers in Elder Care | Empirical | Autonomy, trust, |
| 118 | **2019** | **Huisman** | Two-Year Use of Care Robot Zora in Dutch Nursing Homes: An Evaluation Study dagger | Empirical | Human vs. artificial |
| 119 | **2019** | **Law** | Developing assistive robots for people with mild cognitive impairment and mild dementia: A qualitative study with older adults and experts in aged care | Empirical | Human vs. artificial |
| 120 | **2019** | **Niemlä** | Towards Ethical Guidelines of Using Telepresence Robots in Residential Care | Empirical | Privacy, autonomy, responsibility, human vs. artificial |
| 121 | **2019** | **Pol** | Effectiveness of sensor monitoring in a rehabilitation programme for older patients after hip fracture: A three-arm stepped wedge randomised trial | Empirical | None |
| 122 | **2019** | **Ropero** | LARES: An AI-based teleassistance system for emergency home monitoring | Empirical | Autonomy (deception) |
| 123 | **2019** | **Rostill** | Technology-integrated dementia care: trial results | Empirical | Responsibility |
| 124 | **2019** | **Sanchez** | Older people’s attitudes and perspectives of welfare technology in Norway | Qualitative | Privacy, autonomy, human vs. artificial |
| 125 | **2019** | **Tang** | An IoMT-based geriatric care management system for achieving smart health in nursing homes | Experimental | None |
| 126 | **2020** | **Airola** | Domestication of a Robotic Medication-Dispensing Service Among Older People in Finnish Lapland | Qualitative | Privacy |
| 127 | **2020** | **Arthanat** | Caregiver perspectives on a smart home-based socially assistive robot for individuals with Alzheimer’s disease and related dementia | Qualitative | Privacy, trust |
| 128 | **2020** | **Bankole** | BESI: Behavioral and Environmental Sensing and Intervention for Dementia Caregiver Empowerment—Phases 1 and 2 | Mixed methods | Privacy |
| 129 | **2020** | **Coşar** | ENRICHME: Perception and Interaction of an Assistive Robot for the Elderly at Home | Mixed methods | None |
| 130 | **2020** | **Dupuy** | Effects of an assisted living platform amongst frail older adults and their caregivers: 6 months vs. 9 months follow-up across a pilot field study | experimental | None |
| 131 | **2020** | **Easton-Garrett** | Utilizing artificial intelligence for falls management in memory care | Experimental | informed consent – autonomy |
| 132 | **2020** | **Geier** | How nurses assess telepresence systems in outpatient care. A qualitative study | qualitative | Privacy, human vs. artificial |
| 133 | **2020** | **Holthe** | A critical occupational perspective on user engagement of older adults in an assisted living facility in technology research over three years | Mixed methods | Ageism |
| 134 | **2020** | **Hunter** | Issues associated with the management and governance of sensor data and information to assist aging in place: Focus group study with health care professionals | qualitative | Privacy, autonomy ( + informed consent) |
| 135 | **2020** | **Jøranson** | Effects on sleep from group activity with a robotic seal for nursing home residents with dementia: a cluster randomized controlled trial | Experimental | None |
| 136 | **2020** | **Kleiven** | Health professionals' experiences with the implementation of a digital medication dispenser in home care services- A qualitative study | qualitative | Responsibility, trust, human vs. artificial |
| 137 | **2020** | **Lee** | Toward a Sociable and Dependable Elderly Care Robot: Design, Implementation and User Study | Mixed methods | None |
| 138 | **2020** | **Lussier** | Integrating an Ambient Assisted Living monitoring system into clinical decision-making in home care: An embedded case study | Mixed methods | Responsibility, human vs. artificial |
| 139 | **2020** | **Melkas** | Impacts of robot implementation on care personnel and clients in elderly-care institutions | Mixed methods | Responsibility, human vs. artificial, ageism |
| 140 | **2020** | **Mucchiani** | Exploring Low-Cost Mobile Manipulation for Elder Care Within a Community Based Setting | Mixed methods | Human vs. artificial |
| 141 | **2020** | **O’Brien** | Voice‐Controlled Intelligent Personal Assistants to Support Aging in Place | Qualitative | Autonomy, human vs. artificial |
| 142 | **2020a** | **Obayashi** | Can connected technologies improve sleep quality and safety of older adults and care-givers? An evaluation study of sleep monitors and communicative robots at a residential care home in Japan | Mixed methods | Responsibility |
| 143 | **2020b** | **Obayashi** | Pilot and Feasibility Study on Elderly Support Services Using Communicative Robots and Monitoring Sensors Integrated With Cloud Robotics | Mixed methods | Privacy, autonomy, responsibility |
| 144 | **2020** | **Pais** | Evaluation of 1-Year in-Home Monitoring Technology by Home-Dwelling Older Adults, Family Caregivers, and Nurses | Mixed methods | None |
| 145 | **2020** | **Pazhoumand-Dar** | Detecting deviations from activities of daily living routines using kinect depth maps and power consumption data | Quantitative | None |
| 146 | **2020** | **Pu** | The Effect of Using PARO for People Living With Dementia and Chronic Pain: A Pilot Randomized Controlled Trial | Experimental | None |
| 147 | **2020** | **Rawtaer** | Early detection of mild cognitive impairment with in-home sensors to monitor behavior patterns in community-dwelling senior citizens in Singapore: Cross-sectional feasibility study | Experimental | Ageism |
| 148 | **2020** | **Robinson** | Technology for healthy independent living: Creating a tailored in-home sensor system for older adults and family caregivers | Qualitative | Privacy, responsibility, ageism |
| 149 | **2020** | **Salichs** | Mini: A New Social Robot for the Elderly | Quantitative | Autonomy, human vs. artificial |
| 150 | **2020** | **Snyder** | Remote monitoring technologies in dementia care: An interpretative phenomenological analysis of family caregivers’ experiences | Qualitative | Privacy, trust |
| 151 | **2020** | **Suwa** | Exploring perceptions toward home-care robots for older people in Finland, Ireland, and Japan: A comparative questionnaire study | Quantitative | Privacy, autonomy, trust, human vs. artificial |
| 152 | **2020** | **VandeWeerd** | HomeSense: Design of an ambient home health and wellness monitoring platform for older adults | Experimental | None |
| 153 | **2020** | **Verloo** | Perceptions About Technologies That Help Community-Dwelling Older Adults Remain at Home: Qualitative Study | qualitative | Human vs. artificial |
| 154 | **2020** | **Wang** | A Personalized Health Monitoring System for Community-Dwelling Elderly People in Hong Kong: Design, Implementation, and Evaluation Study | quantitative | None |
| 155 | **2020** | **Woods** | Subverting the logics of “smartness” in Singapore: Smart eldercare and parallel regimes of sustainability | qualitative | Human vs. artificial |
| 156 | **2020** | **Yamakazi** | Anxiety Reduction Through Close Communication with Robotic Media in Dementia Patients and Healthy Older Adults | Experimental | None |
