# Supplementary material for: Mapping ethical issues in the use of smart home health technologies to care for older persons: a systematic review
Source: BMC Med Ethics. 2023 Mar 29;24:24. doi: 10.1186/s12910-023-00898-w (PMC10061702; doi:10.1186/s12910-023-00898-w)
Supplement: Supplementary file 3 — Additional File 3: Appendix part 2 [file 12910_2023_898_MOESM3_ESM.docx]

**Appendix Part 2**

**PROSPERO Registration Name, Number and Date:**

*Name*: "Existing knowledge and ethical concerns associated with the use of smart home health technologies in the care of older persons: a systematic review"

*Number*: CRD42021248543

*Date*: 13.05.2021

**Access to additional material:**

*Review Protocol:* The submitting & first author possesses the review protocol, which can be sent to researchers upon request. Furthermore, the used search strings are documented below.

*Template data collection forms:* The submitting & first author possesses the review protocol, which can be sent to researchers upon request.

*Data extracted from included studies:* The submitting & first author possesses the review protocol, which can be sent to researchers upon request.

*Data used for all analyses:* The submitting & first author possesses the review protocol, which can be sent to researchers upon request.

**Supplementary Table 2: Key search terms and search strategy* §**

| **PPCC** | **Search term** | **Synonyms** |
| --- | --- | --- |
| Population 1 | Older adults | “older adult*” OR “ag*ng” OR “elder*” OR “senior*” OR “geriatr*” OR “gerontolo*” OR “aged person” OR “older person*” OR “elderly people” OR “older parent*” |
| Population 2 | caregivers | “caregiv*” OR “informal caregiv*” OR “unpaid caregiv*” OR “famil* caregiv*” OR “care*” OR “formal caregiv*” OR “professional caregiv*” OR “nurse*” OR “nurse-aid*” OR “physician*” OR “doctor*” OR “spouse*” OR “adult child*” OR “daughter*” OR “wife” OR “husband” OR “son” OR “relative” |
| Context 1 | Smart home technology terminologies | “smart house” OR “welfare technology” OR “smart home” OR “smart technolog*” OR “smart living” OR  “home automation” OR “wireless home automation system*” OR “intelligent living” OR “intelligent building” OR  “domotic*” OR “assistive domotic*” OR  “embedded health system*” OR “ehealth” OR “health monitoring” OR “home-based health technology” OR  “gerontechnology” OR “gerotechnology” OR  “sensors” OR “wearable*” OR “Robotic” OR “Artificial Intelligence in Eldercare” OR “Digital monitor*” OR  “smart technologies to support healthy aging” OR “information technolog* for assisted living at home” OR “home-based assistive technolog*” OR “Ambient Assistive Living” OR  “Intelligent Assistive Technolog*” OR “Intelligent Assistive Device*” OR “Intelligent Assistive Application” |
| Context 2 | Home “home” setting as the individual’s place of residence | “Home care” or “Nursing Home*” or “Independent Living” OR “Home*” OR “house*” OR “homes for the aged” OR “assisted living facilit*” OR “retirement home*” |

* The search terms have been adapted to the relevant database standards. Within PPCC synonyms are linked by Boolean ORs, between PPCC are linked with AND. Other sources: Citation tracking, reading references.

§ **Complete Search String: EMBASE (last tried 25.03.2021)**

Population 1 + Context 1 + Population 2 + Context 2:

('aged'/exp OR 'aging'/exp OR 'geriatrics'/exp OR 'gerontology'/exp OR 'older adult':ti,ab OR 'ag*ng':ti,ab OR 'elder':ti,ab OR 'senior':ti,ab OR 'geriatr*':ti,ab OR 'gerontolo':ti,ab OR 'aged person':ti,ab OR 'old*':ti,ab) AND ('wireless communication'/exp OR 'assistive technology'/exp OR 'telemedicine'/exp OR 'gerontechnology'/exp OR 'smart home'/exp OR 'sensor'/exp OR 'wearable sensor'/exp OR 'wearable computer'/exp OR 'wearable device'/exp OR 'robotics'/de OR 'artificial intelligence'/exp OR 'information technology device'/exp OR 'information technology'/exp OR 'ambient intelligence'/exp OR 'welfare technology':ti,ab OR 'smart home':ti,ab OR 'smart home technolog*':ti,ab OR 'smart living':ti,ab OR 'home automation':ti,ab OR 'wireless home automation system*':ti,ab OR 'intelligent living':ti,ab OR 'intelligent building':ti,ab OR 'domotic*':ti,ab OR 'assistive domotic*':ti,ab OR 'embedded health system*':ti,ab OR 'ehealth':ti,ab OR 'health monitoring':ti,ab OR 'home-based health technology':ti,ab OR 'gerontechnology':ti,ab OR 'gerotechnology':ti,ab OR 'sensors':ti,ab OR 'wearable*':ti,ab OR 'robotic':ti,ab OR 'artificial intelligence in eldercare':ti,ab OR 'digital monitor*':ti,ab OR 'smart technologies to support healthy aging':ti,ab OR 'home-based assistive technolog*':ti,ab OR 'ambient assistive living':ti,ab OR 'intelligent assistive technolog*':ti,ab OR 'intelligent assistive device*':ti,ab OR 'intelligent assistive application':ti,ab) AND ('caregiver'/exp OR 'informal caregiver'/exp OR 'informal caregiving'/exp OR 'family caregiving'/exp OR 'care'/exp OR 'care and caring'/de OR 'nurse'/exp OR 'nursing'/exp OR 'nurse assistant'/exp OR 'physician'/de OR 'spouse'/exp OR 'adult child'/exp OR 'daughter'/exp OR 'wife'/exp OR 'husband'/exp OR 'son'/exp OR 'relative'/exp OR 'caregiv*':ab,ti OR 'informal caregiv*':ab,ti OR 'unpaid caregiv*':ab,ti OR 'famil* caregiv*':ab,ti OR 'care*':ab,ti OR 'formal caregiv*':ab,ti OR 'professional caregiv*':ab,ti OR 'nurse*':ab,ti OR 'nurse-aid*':ab,ti OR 'physician*':ab,ti OR 'doctor*':ab,ti OR spouse*:ab,ti OR 'adult child*':ab,ti OR 'daughter*':ab,ti OR 'wife':ab,ti OR 'husband':ab,ti OR 'son':ab,ti OR 'relative':ab,ti) AND ('home care'/exp OR 'nursing home'/exp OR 'independent living'/exp OR 'home'/exp OR 'house'/exp OR 'household'/exp OR 'home for the aged'/exp OR 'assisted living facility'/exp OR 'home care':ab,ti OR 'nursing home*':ab,ti OR 'independent living':ab,ti OR 'home*':ab,ti OR 'house*':ab,ti OR 'homes for the aged':ab,ti OR 'assisted living facilit*':ab,ti OR 'retirement home*':ab,ti) AND [embase]/lim AND [2000-2020]/py

§ **Complete Search String: MEDLINE (last tried 29.03.2021)**

((((("Aged"[Mesh]) OR "Aging"[Mesh] OR "Geriatrics"[Mesh]) OR ("older adult*"[Title/Abstract] OR "ag*ng"[Title/Abstract] OR "elder*"[Title/Abstract] OR "senior*"[Title/Abstract] OR "geriatr*"[Title/Abstract] OR "gerontolo*"[Title/Abstract] OR "aged person"[Title/Abstract] OR "older person*"[Title/Abstract] OR "elderly people"[Title/Abstract] OR "older parent*"[Title/Abstract]) AND ((medline[Filter]) AND (2000:2020[pdat]))) AND (("Ambient Intelligence"[MeSH Terms] OR "Self-Help Devices"[MeSH Terms:noexp] OR "Telemedicine"[MeSH Terms] OR "Wearable Electronic Devices"[MeSH Terms] OR "Robotics"[MeSH Terms:noexp] OR "Artificial Intelligence"[MeSH Terms]) OR ("smart house"[Title/Abstract] OR "welfare technology"[Title/Abstract] OR "smart home"[Title/Abstract] OR "smart technolog*"[Title/Abstract] OR "smart living"[Title/Abstract] OR "home automation"[Title/Abstract] OR "wireless home automation system*"[Title/Abstract] OR "intelligent living"[Title/Abstract] OR "intelligent building"[Title/Abstract] OR "domotic*"[Title/Abstract] OR "assistive domotic*"[Title/Abstract] OR "embedded health system*"[Title/Abstract] OR "ehealth"[Title/Abstract] OR "health monitoring"[Title/Abstract] OR "home-based health technology"[Title/Abstract] OR "gerontechnology"[Title/Abstract] OR "gerotechnology"[Title/Abstract] OR "sensors"[Title/Abstract] OR "wearable*"[Title/Abstract] OR "Robotic"[Title/Abstract] OR "Artificial Intelligence in Eldercare"[Title/Abstract] OR "Digital monitor*"[Title/Abstract] OR "smart technologies to support healthy aging"[Title/Abstract] OR "information technolog* for assisted living at home"[Title/Abstract] OR "home-based assistive technolog*"[Title/Abstract] OR "Ambient Assistive Living"[Title/Abstract] OR "Intelligent Assistive Technolog*"[Title/Abstract] OR "Intelligent Assistive Device"[Title/Abstract] OR "Intelligent Assistive Application"[Title/Abstract]) AND ((medline[Filter]) AND (2000:2020[pdat])))) AND (("Caregivers"[MeSH Terms] OR "Home Health Nursing"[MeSH Terms] OR "Nurses"[MeSH Terms] OR "Nursing Assistants"[MeSH Terms:noexp] OR "Physicians"[MeSH Terms:noexp] OR "Spouses"[MeSH Terms] OR "Adult Children"[MeSH Terms] OR "Family"[MeSH Terms:noexp] OR "caregiv*"[Title/Abstract] OR "informal caregiv*"[Title/Abstract] OR "unpaid caregiv*"[Title/Abstract] OR "famil* caregiv*"[Title/Abstract] OR "care*"[Title/Abstract] OR "formal caregiv*"[Title/Abstract] OR "professional caregiv*"[Title/Abstract] OR "nurse*"[Title/Abstract] OR "nurse aid*"[Title/Abstract] OR "physician*"[Title/Abstract] OR "doctor*"[Title/Abstract] OR "spouse*"[Title/Abstract] OR "adult child*"[Title/Abstract] OR "daughter*"[Title/Abstract] OR "wife"[Title/Abstract] OR "husband"[Title/Abstract] OR "son"[Title/Abstract] OR "relative"[Title/Abstract]) AND ((medline[Filter]) AND (2000:2020[pdat])))) AND (("Home care"[Title/Abstract] OR "Nursing Home*"[Title/Abstract] OR "Independent Living"[Title/Abstract] OR "Home*"[Title/Abstract] OR "house*"[Title/Abstract] OR "homes for the aged"[Title/Abstract] OR "assisted living facilit*"[Title/Abstract] OR "retirement home*"[Title/Abstract]) OR ("Home Care Services"[Mesh] OR "Home Nursing"[Mesh:NoExp] OR "Nursing Homes"[Mesh] OR "Independent Living"[Mesh] OR "Housing for the Elderly"[Mesh] OR "Homes for the Aged"[Mesh] OR "Assisted Living Facilities"[Mesh]))

§ **Complete Search String: Web of Science (last tried 25.03.2021)**

TS=(("older adult*” OR “ag*ng” OR “elder*” OR “senior*” OR “geriatr*” OR “gerontolo*” OR “aged person” OR “older person*” OR “elderly people” OR “older parent*”) AND (“smart house” OR “welfare technology” OR “smart home” OR “smart technolog*” OR “smart living” OR “home automation” OR “wireless home automation system*” OR “intelligent living” OR “intelligent building” OR “domotic*” OR “assistive domotic*” OR “embedded health system*” OR “ehealth” OR “health monitoring” OR “home-based health technology” OR “gerontechnology” OR “gerotechnology” OR “sensors” OR “wearable*” OR “Robotic” OR “Artificial Intelligence in Eldercare” OR “Digital monitor*” OR “smart technologies to support healthy aging” OR “information technolog* for assisted living at home” OR “home-based assistive technolog*” OR “Ambient Assistive Living” OR “Intelligent Assistive Technolog*” OR “Intelligent Assistive Device*” OR “Intelligent Assistive Application”) AND ("caregiv*" OR "informal caregiv*" OR "unpaid caregiv*" OR "famil* caregiv*" OR "care*" OR "formal caregiv*" OR "professional caregiv*" OR "nurse*" OR "nurse-aid*" OR "physician*" OR "doctor*" OR "spouse*" OR "adult child*" OR "daughter*" OR "wife" OR "husband" OR "son" OR "relative") AND (“Home care” or “Nursing Home*” or “Independent Living” or “Home*” OR “house*” OR “homes for the aged” OR “assisted living facilit*” OR “retirement home*”)) Timespan=2000-2020

§ **Complete Search String: PsychInfo (last tried 29.03.2021)**

((older adult* or ag*ng or elder* or senior* or geriatr* or gerontolo* or aged person or older person* or elderly people or older parent*).ab.) OR (older adult* or ag*ng or elder* or senior* or geriatr* or gerontolo* or aged person or older person* or elderly people or older parent*).ti. OR (exp Aging/ or exp Geriatrics/ or exp Older Adulthood/ or exp Gerontology/ or exp Elder Care/ or exp Geriatric Patients/).sh.

**AND**

(exp Wearable Devices/ OR exp assistive technology/ OR exp Robotics) OR (smart house OR welfare technology OR smart home OR smart technolog* OR smart living OR home automation OR wireless home automation system* OR intelligent living OR intelligent building OR domotic* OR assistive domotic* OR embedded health system* OR ehealth OR health monitoring OR home-based health technology OR gerontechnology OR gerotechnology OR sensors OR wearable* OR Robotic OR Artificial Intelligence in Eldercare OR Digital monitor* OR smart technologies to support healthy aging OR information technolog* for assisted living at home OR home-based assistive technolog* OR Ambient Assistive Living OR Intelligent Assistive Technolog* OR Intelligent Assistive Device* OR Intelligent Assistive Application).ab. OR

(smart house OR welfare technology OR smart home OR smart technolog* OR smart living OR home automation OR wireless home automation system* OR intelligent living OR intelligent building OR domotic* OR assistive domotic* OR embedded health system* OR ehealth OR health monitoring OR home-based health technology OR gerontechnology OR gerotechnology OR sensors OR wearable* OR Robotic OR Artificial Intelligence in Eldercare OR Digital monitor* OR smart technologies to support healthy aging OR information technolog* for assisted living at home OR home-based assistive technolog* OR Ambient Assistive Living OR Intelligent Assistive Technolog* OR Intelligent Assistive Device* OR Intelligent Assistive Application).ti.

**AND**

(exp Caregivers/ OR exp Caregiving/ OR exp Elder Care/ OR exp Nursing/ OR exp Nurses/ OR exp Physicians/ OR exp Spouses/ OR exp Adult Offspring/ OR exp Daughters/ OR exp Wives/ OR exp Husbands/ OR exp Sons/ OR exp Family Members/) OR

(caregiv* OR informal caregiv* OR unpaid caregiv* OR famil* caregiv* OR care* OR formal caregiv* OR professional caregiv* OR nurse* OR nurse-aid* OR physician* OR doctor* OR spouse* OR adult child* OR daughter* OR wife OR husband OR son OR relative).ab. OR

(caregiv* OR informal caregiv* OR unpaid caregiv* OR famil* caregiv* OR care* OR formal caregiv* OR professional caregiv* OR nurse* OR nurse-aid* OR physician* OR doctor* OR spouse* OR adult child* OR daughter* OR wife OR husband OR son OR relative).ti.

**AND**

(exp home care/ OR nursing homes/ OR self-care skills/ OR exp home environment/ OR assisted living/ OR retirement communities/) OR (Home care or Nursing Home* or Independent Living or Home* or house* or homes for the aged or assisted living facilit* or retirement home*).ab. OR (Home care or Nursing Home* or Independent Living or Home* or house* or homes for the aged or assisted living facilit* or retirement home*).ti.

§ **Complete Search String: CINAHL (last tried 29.03.2021)**

(AB ( “older adult*” OR “ag*ng” OR “elder*” OR “senior*” OR “geriatr*” OR “gerontolo*” OR “aged person” OR “older person*” OR “elderly people” OR “older parent*” ) OR TI ( (“older adult*” OR “ag*ng” OR “elder*” OR “senior*” OR “geriatr*” OR “gerontolo*” OR “aged person” OR “older person*” OR “elderly people” OR “older parent*”) ) OR ( (MH "Aged+") OR (MH "Aging+") OR (MH "Geriatrics") OR (MH "Gerontologic Care") OR (MH "Older Adult Care (Saba CCC)") )) AND (( (MH "Assistive Technology Services") OR (MH "Assistive Technology") OR (MH "Home Health Care Information Systems") OR (MH "Assistive Technology Devices") OR (MH "Telehealth+") OR (MH "Monitoring, Physiologic+") OR (MH "Wearable Sensors+") OR (MH "Exoskeleton Devices") OR (MH "Robotics+") OR (MH "Healthy Aging") OR (MH "Assistive Device Therapy (Saba CCC)" OR (MH "Artificial Intelligence+")) ) OR TI ( (“smart house” OR “welfare technology” OR “smart home” OR “smart technolog*” OR “smart living” OR “home automation” OR “wireless home automation system*” OR “intelligent living” OR “intelligent building” OR “domotic*” OR “assistive domotic*” OR “embedded health system*” OR “ehealth” OR “health monitoring” OR “home-based health technology” OR “gerontechnology” OR “gerotechnology” OR “sensors” OR “wearable*” OR “Robotic” OR “Artificial Intelligence in Eldercare” OR “Digital monitor*” OR “smart technologies to support healthy aging” OR “information technolog* for assisted living at home” OR “home-based assistive technolog*” OR “Ambient Assistive Living” OR “Intelligent Assistive Technolog*” OR “Intelligent Assistive Device*” OR “Intelligent Assistive Application”) ) OR AB ( (“smart house” OR “welfare technology” OR “smart home” OR “smart technolog*” OR “smart living” OR “home automation” OR “wireless home automation system*” OR “intelligent living” OR “intelligent building” OR “domotic*” OR “assistive domotic*” OR “embedded health system*” OR “ehealth” OR “health monitoring” OR “home-based health technology” OR “gerontechnology” OR “gerotechnology” OR “sensors” OR “wearable*” OR “Robotic” OR “Artificial Intelligence in Eldercare” OR “Digital monitor*” OR “smart technologies to support healthy aging” OR “information technolog* for assisted living at home” OR “home-based assistive technolog*” OR “Ambient Assistive Living” OR “Intelligent Assistive Technolog*” OR “Intelligent Assistive Device*” OR “Intelligent Assistive Application”) )) AND (AB ( ("caregiv*" OR "informal caregiv*" OR "unpaid caregiv*" OR "famil* caregiv*" OR "care*" OR "formal caregiv*" OR "professional caregiv*" OR "nurse*" OR "nurse-aid*" OR "physician*" OR "doctor*" OR "spouse*" OR "adult child*" OR "daughter*" OR "wife" OR "husband" OR "son" OR "relative”) ) OR TI ( ("caregiv*" OR "informal caregiv*" OR "unpaid caregiv*" OR "famil* caregiv*" OR "care*" OR "formal caregiv*" OR "professional caregiv*" OR "nurse*" OR "nurse-aid*" OR "physician*" OR "doctor*" OR "spouse*" OR "adult child*" OR "daughter*" OR "wife" OR "husband" OR "son" OR "relative”) ) OR ( (MH "Caregivers") OR (MH "Family Caregiver Status (Iowa NOC)") OR (MH "Nurses+") OR (MH "Nursing Assistants") OR (MH "Home Health Aides") OR (MH "Physicians") OR (MH "Sons") OR (MH "Spouses") OR (MH "Daughters") OR (MH "Adult Children") OR (MH "Extended Family") )) AND (AB ( (“Home care” or “Nursing Home*” or “Independent Living” or “Home*” OR “house*” OR “homes for the aged” OR “assisted living facilit*” OR “retirement home*”) ) OR TI ( (“Home care” or “Nursing Home*” or “Independent Living” or “Home*” OR “house*” OR “homes for the aged” OR “assisted living facilit*” OR “retirement home*”) ) OR ( (MH "Home Health Care+") OR (MH "Home Nursing") OR (MH "Nursing Homes+") OR (MH "Community Living+") OR (MH "Housing for the Elderly") OR (MH "Assisted Living") ))

§ **Complete Search String: SocINDEX (last tried 29.03.2021)**

TI ( “older adult*” OR “ag*ng” OR “elder*” OR “senior*” OR “geriatr*” OR “gerontolo*” OR “aged person” OR “older person*” OR “elderly people” OR “older parent*” ) OR AB ( “older adult*” OR “ag*ng” OR “elder*” OR “senior*” OR “geriatr*” OR “gerontolo*” OR “aged person” OR “older person*” OR “elderly people” OR “older parent*” ) OR ( (DE "AGING") OR (DE "ACTIVE aging") OR (DE "GERONTOLOGY") OR (DE "OLDER people") OR (DE "GERIATRICS") OR (DE "GERONTOLOGY") OR (DE "SOCIAL gerontology") OR (DE "OLDER parents") )

**AND**

AB ( (“smart house” OR “welfare technology” OR “smart home” OR “smart technolog*” OR “smart living” OR “home automation” OR “wireless home automation system*” OR “intelligent living” OR “intelligent building” OR “domotic*” OR “assistive domotic*” OR “embedded health system*” OR “ehealth” OR “health monitoring” OR “home-based health technology” OR “gerontechnology” OR “gerotechnology” OR “sensors” OR “wearable*” OR “Robotic” OR “Artificial Intelligence in Eldercare” OR “Digital monitor*” OR “smart technologies to support healthy aging” OR “information technolog* for assisted living at home” OR “home-based assistive technolog*” OR “Ambient Assistive Living” OR “Intelligent Assistive Technolog*” OR “Intelligent Assistive Device*” OR “Intelligent Assistive Application”) ) OR TI ( (“smart house” OR “welfare technology” OR “smart home” OR “smart technolog*” OR “smart living” OR “home automation” OR “wireless home automation system*” OR “intelligent living” OR “intelligent building” OR “domotic*” OR “assistive domotic*” OR “embedded health system*” OR “ehealth” OR “health monitoring” OR “home-based health technology” OR “gerontechnology” OR “gerotechnology” OR “sensors” OR “wearable*” OR “Robotic” OR “Artificial Intelligence in Eldercare” OR “Digital monitor*” OR “smart technologies to support healthy aging” OR “information technolog* for assisted living at home” OR “home-based assistive technolog*” OR “Ambient Assistive Living” OR “Intelligent Assistive Technolog*” OR “Intelligent Assistive Device*” OR “Intelligent Assistive Application”) ) OR (DE "TECHNOLOGY & older people")

**AND**

AB ( ("caregiv*" OR "informal caregiv*" OR "unpaid caregiv*" OR "famil* caregiv*" OR "care*" OR "formal caregiv*" OR "professional caregiv*" OR "nurse*" OR "nurse-aid*" OR "physician*" OR "doctor*" OR "spouse*" OR "adult child*" OR "daughter*" OR "wife" OR "husband" OR "son" OR "relative”) ) OR TI ( ("caregiv*" OR "informal caregiv*" OR "unpaid caregiv*" OR "famil* caregiv*" OR "care*" OR "formal caregiv*" OR "professional caregiv*" OR "nurse*" OR "nurse-aid*" OR "physician*" OR "doctor*" OR "spouse*" OR "adult child*" OR "daughter*" OR "wife" OR "husband" OR "son" OR "relative”) ) OR ( (MH "Caregivers") OR (MH "Family Caregiver Status (Iowa NOC)") OR (MH "Nurses+") OR (MH "Nursing Assistants") OR (MH "Home Health Aides") OR (MH "Physicians") OR (MH "Sons") OR (MH "Spouses") OR (MH "Daughters") OR (MH "Adult Children") OR (MH "Extended Family") )

**AND**

AB ( (“Home care” or “Nursing Home*” or “Independent Living” or “Home*” OR “house*” OR “homes for the aged” OR “assisted living facilit*” OR “retirement home*”) ) OR TI ( (“Home care” or “Nursing Home*” or “Independent Living” or “Home*” OR “house*” OR “homes for the aged” OR “assisted living facilit*” OR “retirement home*”) ) OR ( (MH "Home Health Care+") OR (MH "Home Nursing") OR (MH "Nursing Homes+") OR (MH "Community Living+") OR (MH "Housing for the Elderly") OR (MH "Assisted Living") )

§ **Complete Search String: SCOPUS (last tried 30.03.2021)**

( ( TITLE ( ( "older adult*" OR "ag*ng" OR "elder*" OR "senior*" OR "geriatr*" OR "gerontolo*" OR "aged person" OR "older person*" OR "elderly people" OR "older parent*" ) ) OR ABS ( ( "older adult*" OR "ag*ng" OR "elder*" OR "senior*" OR "geriatr*" OR "gerontolo*" OR "aged person" OR "older person*" OR "elderly people" OR "older parent*" ) ) ) ) AND ( ( ABS ( ( "smart house" OR "welfare technology" OR "smart home" OR "smart technolog*" OR "smart living" OR "home automation" OR "wireless home automation system*" OR "intelligent living" OR "intelligent building" OR "domotic*" OR "assistive domotic*" OR "embedded health system*" OR "ehealth" OR "health monitoring" OR "home-based health technology" OR "gerontechnology" OR "gerotechnology" OR "sensors" OR "wearable*" OR "Robotic" OR "Artificial Intelligence in Eldercare" OR "Digital monitor*" OR "smart technologies to support healthy aging" OR "information technolog* for assisted living at home" OR "home-based assistive technolog*" OR "Ambient Assistive Living" OR "Intelligent Assistive Technolog*" OR "Intelligent Assistive Device*" OR "Intelligent Assistive Application" ) ) OR TITLE ( ( "smart house" OR "welfare technology" OR "smart home" OR "smart technolog*" OR "smart living" OR "home automation" OR "wireless home automation system*" OR "intelligent living" OR "intelligent building" OR "domotic*" OR "assistive domotic*" OR "embedded health system*" OR "ehealth" OR "health monitoring" OR "home-based health technology" OR "gerontechnology" OR "gerotechnology" OR "sensors" OR "wearable*" OR "Robotic" OR "Artificial Intelligence in Eldercare" OR "Digital monitor*" OR "smart technologies to support healthy aging" OR "information technolog* for assisted living at home" OR "home-based assistive technolog*" OR "Ambient Assistive Living" OR "Intelligent Assistive Technolog*" OR "Intelligent Assistive Device*" OR "Intelligent Assistive Application" ) ) ) AND PUBYEAR > 1999 AND PUBYEAR < 2021 ) AND ( ( TITLE ( ( "caregiv*" OR "informal caregiv*" OR "unpaid caregiv*" OR "famil* caregiv*" OR "care*" OR "formal caregiv*" OR "professional caregiv*" OR "nurse*" OR "nurse-aid*" OR "physician*" OR "doctor*" OR "spouse*" OR "adult child*" OR "daughter*" OR "wife" OR "husband" OR "son" OR "relative" ) ) OR ABS ( ( "caregiv*" OR "informal caregiv*" OR "unpaid caregiv*" OR "famil* caregiv*" OR "care*" OR "formal caregiv*" OR "professional caregiv*" OR "nurse*" OR "nurse-aid*" OR "physician*" OR "doctor*" OR "spouse*" OR "adult child*" OR "daughter*" OR "wife" OR "husband" OR "son" OR "relative" ) ) ) AND PUBYEAR > 1999 AND PUBYEAR < 2021 ) AND ( ( TITLE ( ( "Home care" OR "Nursing Home*" OR "Independent Living" OR "Home*" OR "house*" OR "homes for the aged" OR "assisted living facilit*" OR "retirement home*" ) ) OR ABS ( ( "Home care" OR "Nursing Home*" OR "Independent Living" OR "Home*" OR "house*" OR "homes for the aged" OR "assisted living facilit*" OR "retirement home*" ) ) ) AND PUBYEAR > 1999 AND PUBYEAR < 2021 ) AND ( LIMIT-TO ( PUBYEAR , 2020 ) OR LIMIT-TO ( PUBYEAR , 2019 ) OR LIMIT-TO ( PUBYEAR , 2018 ) OR LIMIT-TO ( PUBYEAR , 2017 ) OR LIMIT-TO ( PUBYEAR , 2016 ) OR LIMIT-TO ( PUBYEAR , 2015 ) OR LIMIT-TO ( PUBYEAR , 2014 ) OR LIMIT-TO ( PUBYEAR , 2013 ) OR LIMIT-TO ( PUBYEAR , 2012 ) OR LIMIT-TO ( PUBYEAR , 2011 ) OR LIMIT-TO ( PUBYEAR , 2010 ) OR LIMIT-TO ( PUBYEAR , 2009 ) OR LIMIT-TO ( PUBYEAR , 2008 ) OR LIMIT-TO ( PUBYEAR , 2007 ) OR LIMIT-TO ( PUBYEAR , 2006 ) OR LIMIT-TO ( PUBYEAR , 2005 ) OR LIMIT-TO ( PUBYEAR , 2004 ) OR LIMIT-TO ( PUBYEAR , 2003 ) OR LIMIT-TO ( PUBYEAR , 2002 ) OR LIMIT-TO ( PUBYEAR , 2001 ) OR LIMIT-TO ( PUBYEAR , 2000 ) )

§ **Complete Search String: PhilPapers (last tried 30.03.2021)**

TI ( (“older adult*” OR “ag*ng” OR “elder*” OR “senior*” OR “geriatr*” OR “gerontolo*” OR “aged person” OR “older person*” OR “elderly people” OR “older parent*”) ) OR AB ( (“older adult*” OR “ag*ng” OR “elder*” OR “senior*” OR “geriatr*” OR “gerontolo*” OR “aged person” OR “older person*” OR “elderly people” OR “older parent*”) ) OR ( (ZU "aging") or (ZU "old age") or (ZU "elderly") or (ZU "seniors") or (ZU "geriatrics") or (ZU "gerontology") )

**AND**

TI ( (“smart house” OR “welfare technology” OR “smart home” OR “smart technolog*” OR “smart living” OR “home automation” OR “wireless home automation system*” OR “intelligent living” OR “intelligent building” OR “domotic*” OR “assistive domotic*” OR “embedded health system*” OR “ehealth” OR “health monitoring” OR “home-based health technology” OR “gerontechnology” OR “gerotechnology” OR “sensors” OR “wearable*” OR “Robotic” OR “Artificial Intelligence in Eldercare” OR “Digital monitor*” OR “smart technologies to support healthy aging” OR “information technolog* for assisted living at home” OR “home-based assistive technolog*” OR “Ambient Assistive Living” OR “Intelligent Assistive Technolog*” OR “Intelligent Assistive Device*” OR “Intelligent Assistive Application”) ) OR AB ( (“smart house” OR “welfare technology” OR “smart home” OR “smart technolog*” OR “smart living” OR “home automation” OR “wireless home automation system*” OR “intelligent living” OR “intelligent building” OR “domotic*” OR “assistive domotic*” OR “embedded health system*” OR “ehealth” OR “health monitoring” OR “home-based health technology” OR “gerontechnology” OR “gerotechnology” OR “sensors” OR “wearable*” OR “Robotic” OR “Artificial Intelligence in Eldercare” OR “Digital monitor*” OR “smart technologies to support healthy aging” OR “information technolog* for assisted living at home” OR “home-based assistive technolog*” OR “Ambient Assistive Living” OR “Intelligent Assistive Technolog*” OR “Intelligent Assistive Device*” OR “Intelligent Assistive Application”) ) OR ( (ZU "wireless sensor network") or (ZU "assistive technology") or (ZU "sensor") or (ZU "wearable") or (ZU "wearable technology") or (ZU "robotics") or (ZU "artificial intelligence") or (ZU "ambient intelligence") )

**AND**

AB ( ("caregiv*" OR "informal caregiv*" OR "unpaid caregiv*" OR "famil* caregiv*" OR "care*" OR "formal caregiv*" OR "professional caregiv*" OR "nurse*" OR "nurse-aid*" OR "physician*" OR "doctor*" OR "spouse*" OR "adult child*" OR "daughter*" OR "wife" OR "husband" OR "son" OR "relative”) ) OR TI ( ("caregiv*" OR "informal caregiv*" OR "unpaid caregiv*" OR "famil* caregiv*" OR "care*" OR "formal caregiv*" OR "professional caregiv*" OR "nurse*" OR "nurse-aid*" OR "physician*" OR "doctor*" OR "spouse*" OR "adult child*" OR "daughter*" OR "wife" OR "husband" OR "son" OR "relative”) ) OR ( (ZU "caregiver") or (ZU "caregiving") or (ZU "careprovider") or (ZU "caretaker") or (ZU "care") or (ZU "care giving") or (ZU "nurse") or (ZU "nurse practitioner") or (ZU "physician") or (ZU "spouse") or (ZU "daughter") or (ZU "wife") or (ZU "husband") or (ZU "son") or (ZU "relative") )

**AND**

( (ZU "home care") OR (ZU "nursing home") OR (ZU "independent living") OR (ZU "home") OR (ZU "house") or (ZU "household") or (ZU "houses") or (ZU "housing") ) OR ( TI ( (“Home care” or “Nursing Home*” or “Independent Living” or “Home*” OR “house*” OR “homes for the aged” OR “assisted living facilit*” OR “retirement home*”) ) ) OR ( AB ( (“Home care” or “Nursing Home*” or “Independent Living” or “Home*” OR “house*” OR “homes for the aged” OR “assisted living facilit*” OR “retirement home*”) ) )

§ **Complete Search String: IEEE (last tried 30.03.2021)**

(("Document Title":“older adult” OR “aging” OR "ageing" OR “elder” OR "elderly" OR “senior” OR “geriatric” OR “gerontology” OR “aged person” OR “older person” OR “elderly people” OR “older parent”) OR ("Abstract":“older adult” OR “aging” OR "ageing" OR “elder” OR "elderly" OR “senior” OR “geriatric” OR “gerontology” OR “aged person” OR “older person” OR “elderly people” OR “older parent”))

**AND**

(("Document Title":“smart house” OR “welfare technology” OR “smart home” OR “smart technology" OR "smart technologies" OR “smart living” OR “home automation” OR “wireless home automation system” OR “intelligent living” OR “intelligent building” OR “domotic” OR “assistive domotic” OR “embedded health system” OR “ehealth” OR “health monitoring” OR “home-based health technology” OR “gerontechnology” OR “gerotechnology” OR “sensors” OR “wearable” OR “Robotic” OR “Artificial Intelligence in Eldercare” OR “Digital monitor” OR “smart technologies to support healthy aging” OR “information technology for assisted living at home” OR "information technologies for assisted living at home" OR “home-based assistive technology" OR "home-based assistive technologies" OR “Ambient Assistive Living” OR “Intelligent Assistive Technology” OR "Intelligent Assistive Technologies" OR “Intelligent Assistive Device” OR “Intelligent Assistive Application”) OR ("Abstract":“smart house” OR “welfare technology” OR “smart home” OR “smart technology" OR "smart technologies" OR “smart living” OR “home automation” OR “wireless home automation system” OR “intelligent living” OR “intelligent building” OR “domotic” OR “assistive domotic” OR “embedded health system” OR “ehealth” OR “health monitoring” OR “home-based health technology” OR “gerontechnology” OR “gerotechnology” OR “sensors” OR “wearable” OR “Robotic” OR “Artificial Intelligence in Eldercare” OR “Digital monitor” OR “smart technologies to support healthy aging” OR “information technology for assisted living at home” OR "information technologies for assisted living at home" OR “home-based assistive technology" OR "home-based assistive technologies" OR “Ambient Assistive Living” OR “Intelligent Assistive Technology” OR "Intelligent Assistive Technologies" OR “Intelligent Assistive Device” OR “Intelligent Assistive Application”))

**AND**

(("Document Title":"caregiver" OR "caregiving" OR "informal caregiver" OR "informal caregiving" OR "unpaid caregiver" OR "unpaid caregiving" OR "family caregiver" OR "families caregiver" OR "family caregiving" OR "families caregiving" OR "carer" OR "formal caregiver" OR "formal caregiving" OR "professional caregiver" OR "professional caregiving" OR "nurse" OR "nurse-aid" OR "physician" OR "doctor" OR "spouse" OR "adult child" OR "adult children" OR "daughter" OR "wife" OR "husband" OR "son" OR "relative”) OR ("Abstract":"caregiver" OR "caregiving" OR "informal caregiver" OR "informal caregiving" OR "unpaid caregiver" OR "unpaid caregiving" OR "family caregiver" OR "families caregiver" OR "family caregiving" OR "families caregiving" OR "carer" OR "formal caregiver" OR "formal caregiving" OR "professional caregiver" OR "professional caregiving" OR "nurse" OR "nurse-aid" OR "physician" OR "doctor" OR "spouse" OR "adult child" OR "adult children" OR "daughter" OR "wife" OR "husband" OR "son" OR "relative”))

**AND**

(("Abstract":"Home care” or “Nursing Home” or “Independent Living” or “Home” OR “house” OR “homes for the aged” OR “assisted living facility” OR "assisted living facilities" OR “retirement home”) OR ("Document Title":“Home care” or “Nursing Home” or “Independent Living” or “Home” OR “house” OR “homes for the aged” OR “assisted living facility” OR "assisted living facilities" OR “retirement home”))
